# Imaging the transcriptome

**DOI:** 10.1038/msb.2013.67

**Published:** 2013-11-26

**Authors:** Timothée Lionnet

**Affiliations:** 1 Transcription Imaging Consortium, Howard Hughes Medical Institute, Ashburn, VA, USA

It is well known that genetically identical cells can display a high variability in their gene expression profiles. This phenomenon has a profound impact on a myriad of cellular processes, ranging from differentiation to signaling and drug resistance. Large efforts are therefore currently devoted to understand the mechanistic causes and functional consequences of cell-to-cell variability, often called noise (Lionnet and Singer, 2012).

There are multiple sources of cell-to-cell variability: cell cycle stage, circadian clock, metastable epigenetic states, fluctuations in the concentration of regulatory factors, inhomogeneous microenvironments, or the stochastic nature of the molecular steps involved in gene expression. These factors are often hard to separate experimentally because they might be unknown *a priori* and are often challenging to control: they can range from intracellular concentrations of upstream factors to cell shape or extracellular context.

As most genetic circuits involve a vast number of genes, it has proven extremely useful to study genome-wide transcriptomes in order to understand the determinants of gene expression variability. The first applications of microarray profiling to single cells were demonstrated a decade ago (Klein et al, 2002), and RNAseq-based methods have recently contributed to increase the assay sensitivity. As a result, these technologies can now map expression data onto the full genome and identify splicing variants. However, scaling the number of cells in this type of assay is both expensive and challenging experimentally; it has so far been limited to less than 20 cells (Shalek et al, 2013). In parallel, multiplex single-cell qPCR techniques have also made great progress and can now interrogate a relatively large number of individual cells, at the expense of the number of genes analyzed (up to 100 genes in 1000 cells; Figure 1A; Wills et al, 2013).

Single-molecule mRNA fluorescent *in situ* hybridization (smFISH) is the main approach complementing sequencing and microarray-based techniques (Itzkovitz and van Oudenaarden, 2011). It consists in hybridizing multiple fluorescent DNA probes to a given mRNA species in a fixed biological sample. Individual mRNA molecules appear as individual spots and can be counted using dedicated algorithms. The technique has the advantage of preserving the integrity of the sample, and thus allows capturing a wealth of parameters (e.g., cell shape and location, mRNA spatial distribution (Chou et al, 2013) or the expression pattern of a protein of interest) that are usually lost in techniques based on cDNA libraries. The main limit of smFISH is its modest throughput: the number of genes one can simultaneously image is limited by the number of spectrally separable fluorescent dyes (∼5). Barcoding approaches have increased this number to ∼30 (Lubeck and Cai, 2012), but these numbers remain exceedingly low compared to the tens of thousands genes composing the human genome. Measuring larger number of genes by smFISH has so far only been possible using artificially labeled reporters in bacteria (Taniguchi et al, 2010).

In a recent article, Battich et al (2013) have demonstrated an automated pipeline for smFISH that allowed them to interrogate separately ∼1000 endogenous genes, collecting data from ∼11 000 individual cells for each gene. This experimental tour de force relies on using a variant of smFISH termed ‘branched DNA smFISH' (bDNA smFISH). Instead of directly labeling the transcripts with fluorescently labeled probes, the technology uses a combination of primary, secondary and tertiary probes that hybridize together in order to label each target site on a given mRNA with tens of fluorescent labels (Figure 1B). As a result of the increased signal, fluorescence images could be acquired faster than with traditional smFISH, using only a low-magnification microscope objective. This allowed scanning a cell population faster, resulting in an increase in the throughput of the technique. The sensitivity of the bDNA smFISH rivals that of RNAseq over most of the expression spectrum (the dynamic range of the FISH technique is slightly lower for highly expressed transcripts). Using their unprecedentedly large data sets, the authors tested the statistical requirements of single-cell mRNA counting. They found that for most genes, at least 1000 individual cells should be analyzed to recapitulate the mRNA copy number distribution in a reproducible fashion. This finding will constitute an important standard for the developing field of single-cell transcriptomics.

The main advantage of the approach lies in its image-based nature. Using an integrated image analysis pipeline, the authors were able to extract a battery of spatially resolved measurements inaccessible to cDNA-based transcriptomics techniques. This information is crucial to investigate determinants of cell-to-cell variability; for instance, as biochemical reactions are dependent on factors concentrations rather than numbers, simply knowing the cell volume is important to normalize copy number fluctuations. Furthermore, the authors found that mRNAs sharing statistical and spatiotemporal expression patterns were likely to encode interacting proteins. This finding demonstrates the important role of mRNA (co)localization in gene expression and may suggest a mechanism explaining why functionally related proteins display correlated expression levels, whereas their respective mRNAs levels are essentially uncorrelated (Gandhi et al, 2011).

Image-based, multivariate approaches will play a crucial role in understanding the determinants of cell identity and variability because they are able to collect single-cell transcriptomes along with information about the respective environment, morphology and eventually function of each cell. As smFISH approaches high throughput, these advantages will make it a major tool for understanding the regulation, function and dysfunction of gene expression heterogeneity.

## Figures and Tables

**Figure 1:**
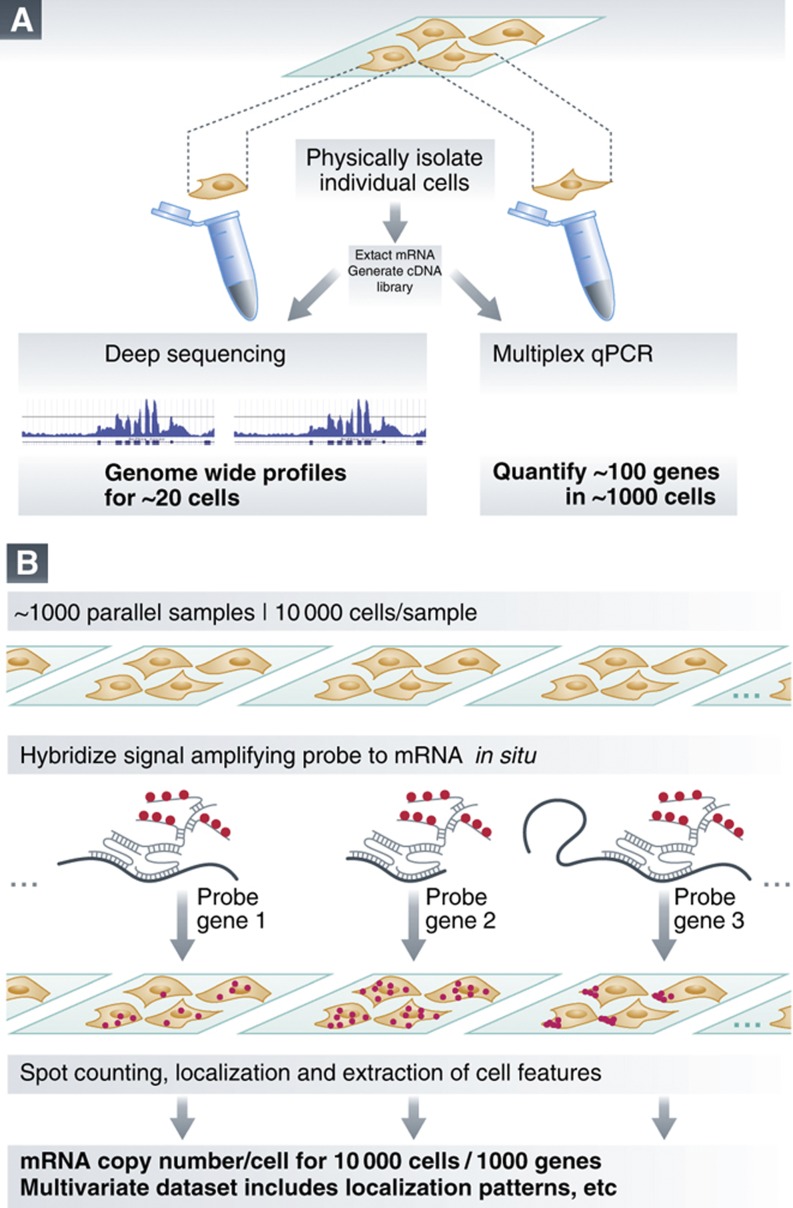
**Techniques used for single-cell transcriptomics**. (**A**) cDNA-based approaches require cell extraction using a micropipette or a sorting device, followed by an amplification step where the RNA content from the single-cell is converted into a cDNA library. Finally the library is analyzed using high throughput sequencing, microarrays or Multiplex qPCR. (**B**) High-throughput FISH. Multiple samples are separately hybridized to a fluorescent probe targeting a given gene. Imaging tens of thousands of cells from each sample yields statistically significant copy number distributions, as well as information about the cell environment, or the localization pattern of the mRNA species (e.g., localization to the edge of the cytosol as in the rightmost sample).
